# Expression of AKRs superfamily and prognostic in human gastric cancer

**DOI:** 10.1097/MD.0000000000033041

**Published:** 2023-02-22

**Authors:** Yujin Zhou, Yi Lin, Wenjing Li, Quan Liu, Hui Gong, Yifan Li, Dixian Luo

**Affiliations:** a NHC Key Laboratory of Metabolic Cardiovascular Diseases Research, Department of Physiology and Pathophysiology, School of Basic Medical Sciences, Ningxia Medical University, Yinchuan, China; b Laboratory Medicine Center of Huazhong University of Science and Technology Union Shenzhen Hospital (Nanshan Hospital), Shenzhen, China.

**Keywords:** AKRs, aldo-keto reductase, gastric cancer, prognosis, transcription factors

## Abstract

The human aldo-keto reductase (AKRs) superfamily is involved in the development of various tumors. However, the different expression patterns of AKRs and their prognostic value in gastric cancer (GC) have not been clarified. In this study, we analyzed the gene expression and gene methylation level of AKRs in GC patients and the survival data and immune infiltration based on AKRs expression, using data from different databases. We found that the expression levels of *AKR1B10, AKR1C1, AKR1C2*, and *AKR7A3* in GC tissues were lower and the expression level of *AKR6A5* was higher in GC tissues than in normal tissue. These differentially expressed genes (*AKR1B10, AKR1C1, AKR1C2, AKR7A3*, and *AKR6A5*) were significantly correlated with the infiltration level. The expression of *SPI1* and *AKR6A5* in GC was positively correlated. Survival analysis showed that GC levels of *AKR6A5* reduced or increased mRNA levels of *AKR7A3*, and *AKR1B10* was expected to have higher overall survival (OS), first progression (FP) survival, and postprogression survival (PPS) rates and a better prognosis. Moreover, the expression of *AKR1B1* was found to be correlated with the staging of GC. The methylation of *AKR6A5 (KCNAB2*) at cg05307871 and cg01907457 was significantly associated with the classification of GC. Meta-analysis and ROC curve analysis show that the expression level of *AKR1B1* and the methylation of cg16156182 (*KCNAB1*), cg11194299 (*KCNAB2*), cg16132520 (*AKR1B1*), and cg13801416 (*AKR1B1*) had a high hazard ratio and a good prognostic value. These data suggest that the expression and methylation of *AKR1B1* and *AKR6A5* are significantly related to the prognosis.

## 1. Introduction

Global morbidity and mortality statistics for 2020 show that gastric cancer (GC) remains prevalent, with close to 1 million new cases and 769,000 deaths in 2020, out of 19.3 million new cancer cases, ranking fifth and fourth in global morbidity and mortality, respectively.^[1]^ In contemporary life, the main treatment methods for GC are chemotherapy, radiotherapy, surgery, and immunotherapy, which have greatly improved the therapeutic effect. Through comprehensive molecular evaluation of 295 samples of GC, 4 molecular subtypes of GC were identified: the Epstein–Barr virus-positive type, with a high frequency of *PIK3CA* gene mutations, extreme hypermethylation of DNA, and amplification of *JAK2, CD274* (also known as *PD-L1*), and *PDCD1LG2* (also known as *PD-L2*) genes; the microsatellite instability type, showing a high mutation rate, including mutations in genes encoding targeted oncogenic signaling proteins such as gastric *CIMP,* and *MHL1* hypermethylation; the genome stable type, characterized by *CDH1, ARID1A*, and *RHOA* gene mutation or RHO family GTP-activated protein gene fusion (*CLDN18-ARHGap* fusion); the chromosomal instability type, characterized by frequent *TP53* gene mutations, significant aneuploidy, increased *EGFR (PY1068*) phosphorylation by *EGFR* gene amplification, and local amplification of the receptor tyrosine kinase gene. Patients are likely to benefit from targeted therapy based on classic clinical prognostic biomarkers identified by typing, such as *EGFR*, receptor tyrosine kinase, and *PD-L1*, which play a role in which patients benefit from targeted therapy.^[2]^

Although major progress has been achieved in the early detection and treatment of GC in the past few decades, because of tumor heterogeneity, at present the prognostic value of biomarkers is still limited, and as a result, there is continuing need to recognize new biomarkers that could serve as prognostic indicators in order to comprehensively improve the prognosis and optimize individualized treatment.

The aldo-keto reductase (AKRs) protein superfamily contains 190 proteins, which are identified by sequence alignment and can be divided into 16 families. AKRs are found in various organisms such as mammals, amphibians, plants, yeasts, protozoans, and bacteria. To date, 15 human AKRs proteins belonged to 3 families AKR1, AKR6, and AKR7, including aldehyde reductase (*AKR1A1*); aldose reductase, and aldose like reductase (*AKR1B1, AKR1B10*, and *AKR1B15*); hydroxysteroid dehydrogenase (*AKR1C1–4*); steroid 5β-reductase (*AKR1D1*); 1,5-dehydration-D-fructose reductase (*AKR1E2*); β-subunits of potassium voltage-gated channels *AKR6A3*(*KCNAB1*), *AKR6A5*(*KCNAB2*), and *AKR6A9*(*KCNAB3*); aflatoxin dialdehyde reductase (*AKR7A3*); and succinate hemi-aldehyde reductase (*AKR7A2*). These proteins belong to 3 families: AKR1, AKR6, and AKR7. Abnormal expression and function of human AKRs can lead to a variety of diseases through the dysregulation of some cellular metabolic pathways, detoxification dysfunction of various endogenous and exogenous carbon-based compounds, or activation of carcinogens. In particular, members of the AKRs family 1 and 7 (AKR1 and AKR7) have been found to be involved in the development of multiple malignancies, such as primary liver cancer, lung cancer, colorectal cancer, and prostate cancer, and breast cancer.

There is only 1 member of the human AKR1A subfamily, *AKR1A1. AKR1A1* mainly acts as a detoxication enzyme.^[3]^ By regulating the activity of endothelial nitric oxide synthase, which is strongly expressed in the proximal renal tubules, *AKR1A1* protects the kidney from acute kidney damage, according to Zhou et al.^[4]^ There are 3 members of the human AKR1B subfamily: *AKR1B1, AKR1B10*, and *AKR1B15*. According to Hojnik et al, *AKR1B1* is a potential predictive biomarker of serous ovarian cancer and is involved in its pathogenesis.^[5]^ *AKR1B10* is overexpressed in human liver cancer, breast cancer, pancreatic cancer, cervical cancer, and smoking-induced lung cancer, and has been identified as a biomarker for nonsmall cell lung cancer (NSCLC). Besides, part of studies has proved that *AKR1B10* is down-regulated in gastrointestinal cancer and several inflammatory bowel diseases, indicating that its low expression is associated with a bad prognosis and reduced survival rates in colorectal cancer and GC patients.^[6,7]^ The steroid hormone metabolism, bile acid biosynthesis, and the metabolism and production of neurosteroids are all crucial processes in which *AKR1C1-4* and *AKR1D1* participate. Changes in the expression of a certain AKR1C gene are associated with the development of prostate, breast, and endometrial cancers_._^[8,9]^ The AKR7 family consists of 2 members, *AKR7A2* and *AKR7A3. AKR7A3* was found to be under expressed in GC. Chow et al also found that *AKR7A3* inhibits liver cancer growth.^[10]^ The human AKR6A subfamily is also known as the voltage-gated potassium channel β-subunit (Kvβ or KCNAB). It includes 3 members: *AKR6A3* (Kvβ1 or *KCNAB1*), *AKR6A5* (Kvβ2 or *KCNAB2*), and *AKR6A9* (Kvβ3 or *KCNAB3*). As the cytoplasmic helper β-subunit of Kv in the Shaker family, AKR6A forms a permanent complex with the pore-forming α-subunit of this channel, which is mainly expressed in the nervous system, heart, and vascular smooth muscle.^[11–14]^ These Kvβ-subunits are particularly important in the cardiovascular system.^[15]^ Obviously, AKRs proteins play roles in various physiological and pathological processes, but the specific mechanism and function of AKRs in GC have not been fully elucidated.

In our study, we used bioinformatics methods to analyze (i) the expression of AKRs in GC and (ii) their correlations with patient prognosis, immune invasion in the tumor microenvironment, and interacting proteins. These results provide an instructive overview of the diagnosis and treatment of GC based on AKRs.

## 2. Materials and Methods

### 2.1. ONCOMINE database analysis

ONCOMINE (https://www.oncomine.org/resource/login.html) is a large cancer gene chip database designed to mine cancer genetic information. We used it to analyze the expression of 15 members of the human AKRs superfamily at the transcriptional level between different cancers and normal controls. The threshold was defined as follows: *P* < .01, fold change ≥ 2, and gene ranking of all.

### 2.2. GEPIA database analysis

Using a common processing pipeline, GEPIA (http://gepia2.cancer-pku.cn/#index) is a visual site that houses a freshly created interactive web server for evaluating the RNA sequencing expression data of 9736 tumors and 8587 normal samples from the TCGA and GTEx projects. Tumor/normal differential expression analysis, analysis by cancer type or pathological stage, patient survival analysis, similar genetic testing, correlation analysis, and dimension reduction analysis are just a few of the adaptable features it offers.

### 2.3. Kaplan–Meier plotter database analysis

With a sample size of over 25,000, the Kaplan–Meier Plotter can assess the relationship between 30,000 genes’ levels of mRNA, miRNA, and protein expression and survival in 21 tumor types, including breast cancer, ovarian cancer, and lung cancer, and GC. For the meta-analysis, the investigation, identification, and confirmation of survival-related molecular markers, the database integrates gene expression data with clinical prognostic values. This database (https://kmplot.com/analysis/index.php?p=service&cancer=gastric) contains the relationships between gene expression and survival of GC patients. Firstly, the patient samples were divided into 2 groups according to the median expression (high expression and low expression), and the overall survival (OS), FP, and PPS of GC patients were analyzed. The HR is shown with 95% confidence intervals (CIs) and log rank *P*-value. The number at risk is indicated below the main plot.

### 2.4. The cancer genome atlas data and cBioPortal

CBioPortal (https://www.cbioportal.org/) enables the exploration, visualization, and analysis of multidimensional cancer genome data, somatic mutations, DNA copy number alterations, mRNA, and miRNA expression, DNA methylation, protein enrichment, and phosphorylated protein enrichment. Based on a stomach adenocarcinoma (STAD) (TCGA, PanCancer Atlas) dataset of 440 cases, including mutations for the AKRs superfamily, putative copy number alterations from genomic identification of significant targets in cancer (GISTIC), mRNA expression Z scores (RNA-seq v.2 RSEM), and protein expression Z scores (reverse phase protein array) were analyzed.

### 2.5. hTFtarget database analysis

hTFtarget (http://bioinfo.life.hust.edu.cn/hTFtarget#!/search) is a comprehensive database of human transcription factors (TFs) target genes. Analysis of TFs binding sites and the influence of TFs binding on genome epigenetic modifications adopted the unified forecasting method to predict the relatively reliable human target genes of TFs and build the open source database of human target genes of TFs. We analyzed gastric tissues through screening for TFs associated with differentially expressed genes in the database.

### 2.6. TIMER database analysis

The TIMER (https://cistrome.shinyapps.io/timer/) database can be used for analyzing immune cell infiltration in tumor tissue, the expression of differentially expressed genes in various cancers, and their correlation with the expression and abundance of immune invasion.

### 2.7. Metascape database analysis

Metascape (http://metascape.org/gp/index.html#/main/step1) is a database for enrichment analysis of biological pathways. It integrates several authoritative functional databases such as GO, KEGG, and UniProt, which can be used to analyze the structure of the protein interaction network of a large number of genes and provide abundant gene annotation and enrichment functions. We conducted a functional enrichment analysis of the top 50 interacting proteins of differentially expressed genes through this website.

### 2.8. STRING and Cytoscape database analysis

STRING (https://cn.string-db.org/) is a database that can search online for known protein interactions. Cytoscape can integrate biological networks with molecular state information such as gene expression and genotype in a visual environment and link these networks to a database of functional annotations. We used the STRING protein interaction database to search for proteins that interact with differential genes and then mapped them using Cytoscape to map protein–protein interaction networks.

### 2.9. Human protein atlas database analysis

The human protein atlas database (https://www.proteinatlas.org/) contains a lot of information about protein research, such as intracellular localization of proteins, expression in human tissues, and expression in tumor tissues.

### 2.10. UCSC database analysis

UCSC Xena (https://xenabrowser.net) allows users to explore functional genomic datasets for analysis of correlations between genomic and/or phenotypic variables. These data are downloaded from the GDC TCGA STAD database, including Gene Expression and Methylation from Gene Expression in Genomic, Illumina Human Methylation 450, Then choose primary_diagnosis. diagnoses in the Phenotypic, find the differences in expression of some loci through a single-sample ANOVA test and select OS and OS time for survival analysis (COX regression) and ROC curve drawing.

### 2.11. Statistical analysis

Firstly, SPSS (IBM SPSS Statistics 25.0) software was used to conduct univariate COX regression analysis on the 15 AKRs genes and methylation sites. Then, the Schoenfeld residual method with *P* < .05 was used to select genes or methylation sites whose PH hypothesis was satisfied by the continuous variable PH hypothesis test. Multivariate COX analysis was performed to construct prognostic gene models, and ROC curves were drawn to determine the prediction accuracy. A *P* value of <.05 was considered statistically significant.

## 3. Results

### 3.1. Transcriptional expression levels of AKRs superfamily genes in GC

**Figure 1.**
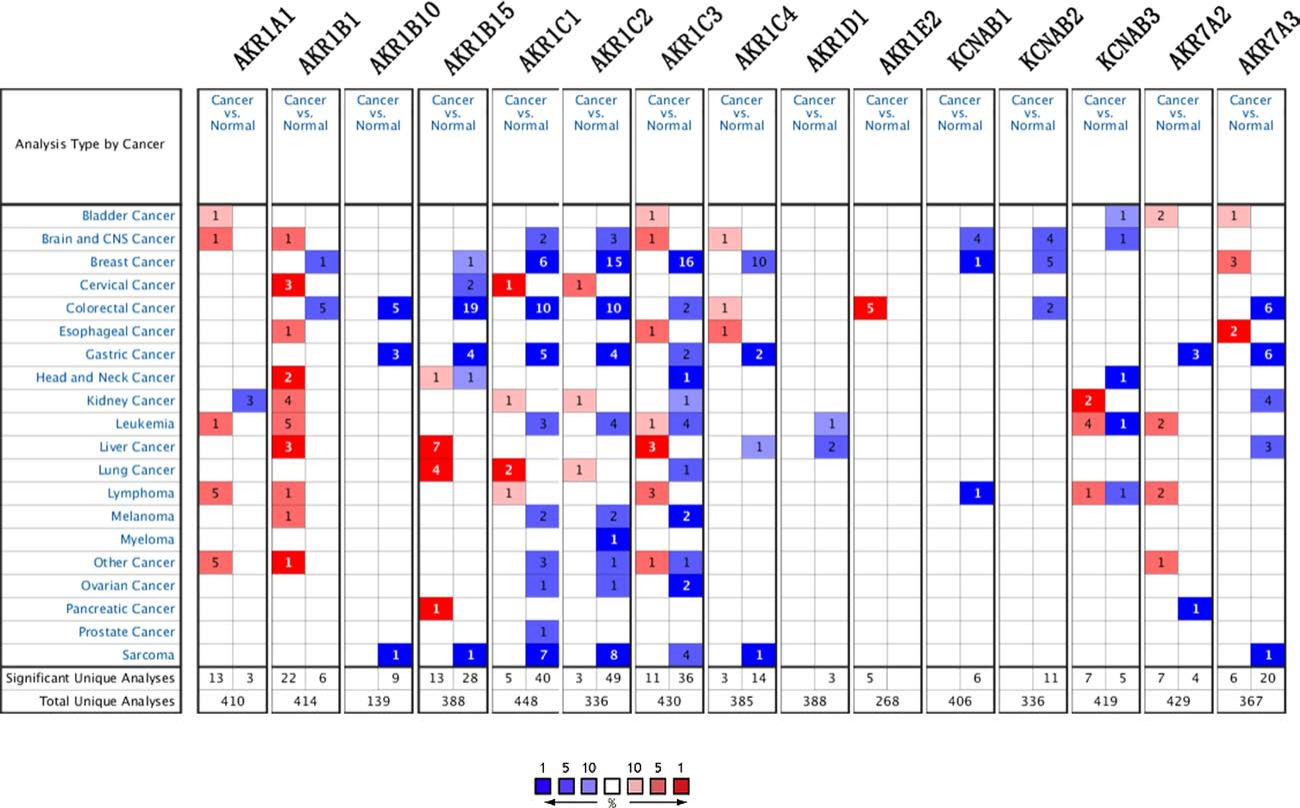
The transcription levels of AKRs genes in different types of cancers (ONCOMINE). AKRs = aldo-keto reductase.

To evaluate the expression levels of AKRs genes in GC and normal samples, we compared their expression in Oncomine database. We found that *AKR1B10, AKR1B15, AKR1C1–4, AKR7A2*, and *AKR7A3* were down-regulated in GC compared with normal tissues (Fig. 1).

### 3.2. Relationship between the clinicopathological characteristics of patients with GC and the AKRs’ mRNA levels

**Figure 2.**
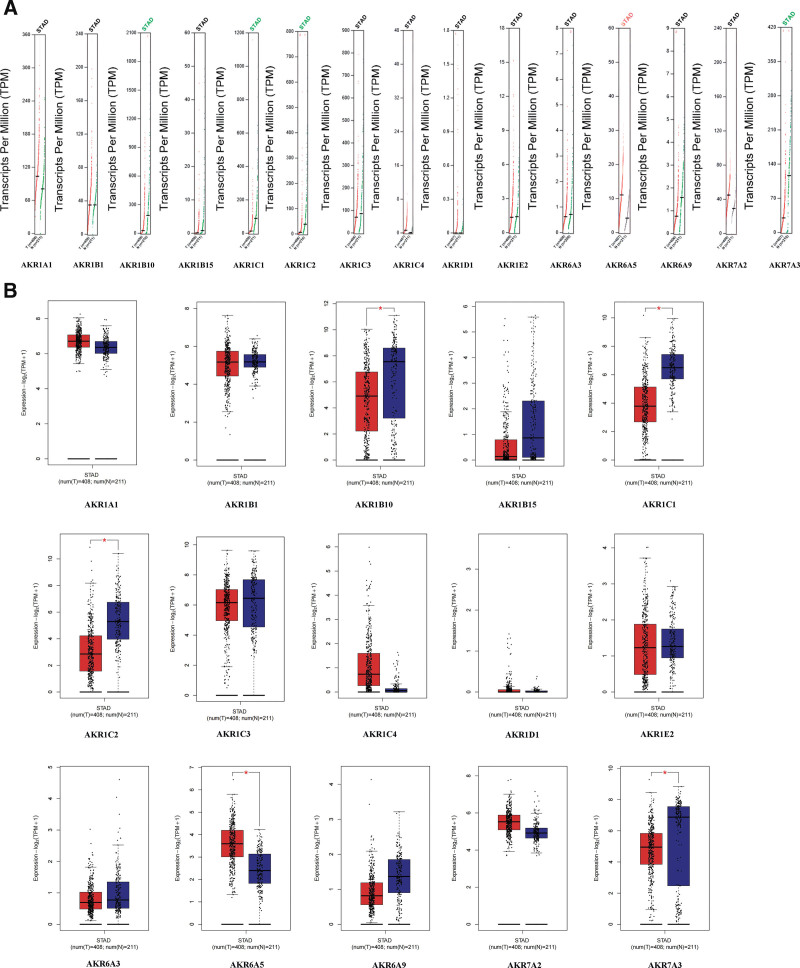
The expression of AKRs in GC (GEPIA). AKRs = aldo-keto reductase, GC = gastric cancer.

Using the Gene Expression Profiling Interactive Analysis (GEPIA) database, we compared the expression of AKRs in GC and normal stomach tissues. Results showed that the expression levels of *AKR1B10, AKR1C1, AKR1C2*, and *AKR7A3* in STAD were lower than those in normal gastric tissues, with the exception that the expression level of *AKR6A5* in GC tissues was higher than that in normal tissues (Fig. 2A and B).

**Figure 3.**
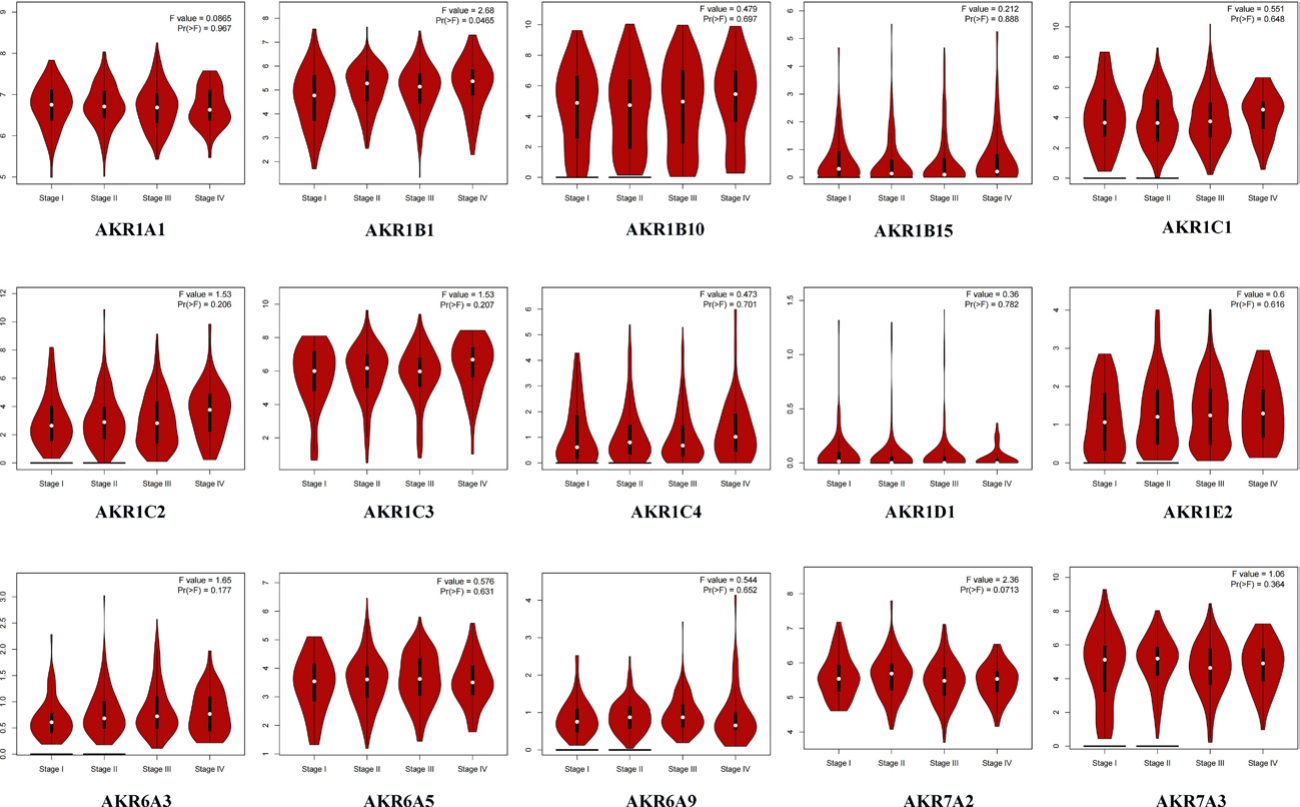
Correlation between AKRs expressions and tumor stages in GC patients (GEPIA). AKRs = aldo-keto reductase, GC = gastric cancer.

The relationship between GC stage and AKRs gene expression was then examined. We found that *AKR1B1* expression was significantly different in different stages of GC (*P* = .0465). However, the gene expression levels of *AKR1A1, AKR1B10, AKR1B15, AKR1C1–4, AKR1D1, AKR1E2, AKR6A3, AKR6A5, AKR6A9, AKR7A2*, and *AKR7A3* were not correlated with GC stages (Fig. 3).

### 3.3. The association between AKRs’ mRNA expression in GC tissues and GC patients’ prognoses

**Figure 4.**
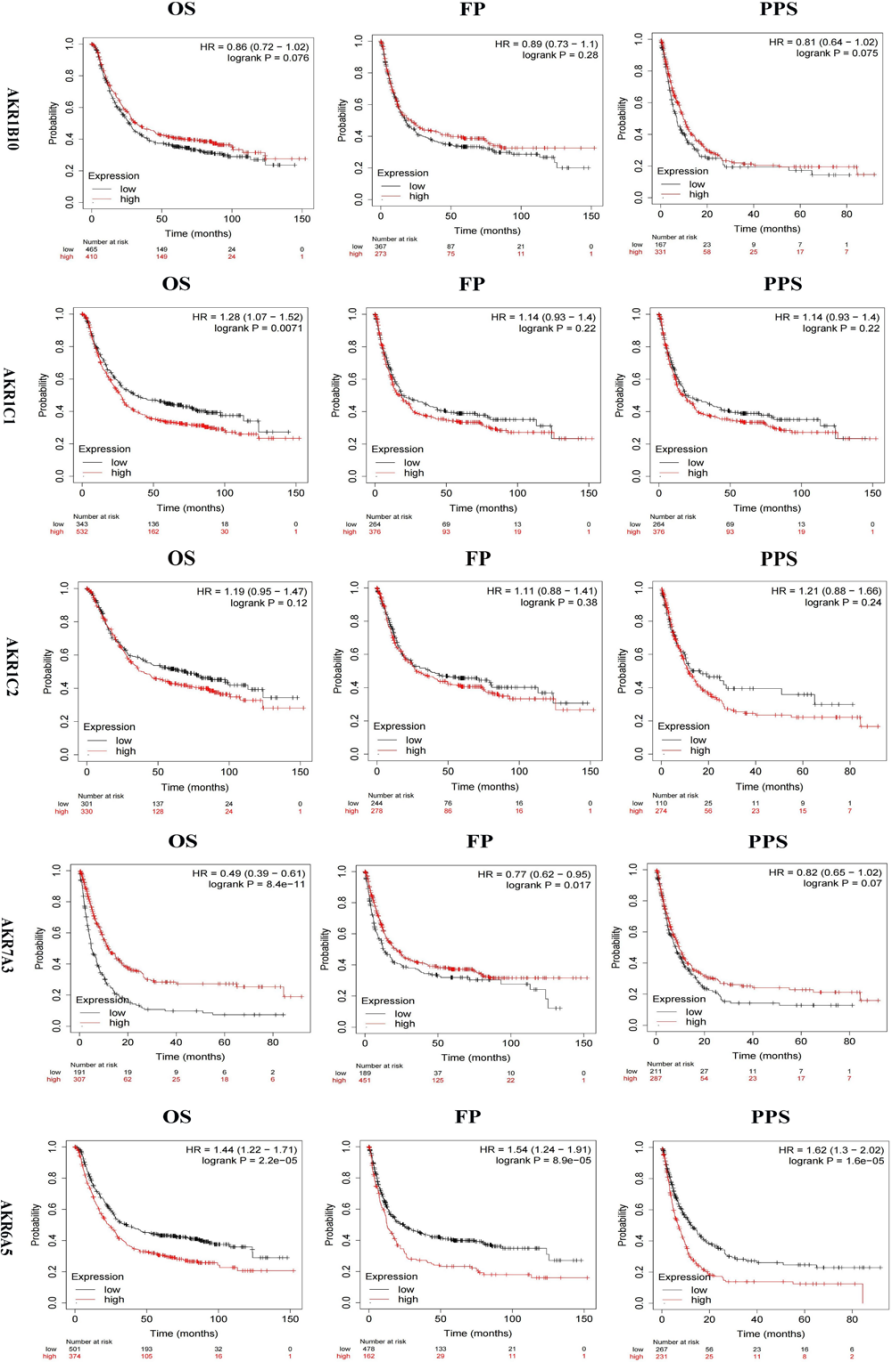
Prognostic value of mRNA levels of differentially expressed genes for GC patients (Kaplan–Meier Plotter). GC = gastric cancer.

We investigated the association between AKRs gene expression and survival using publicly available datasets. A Kaplan–Meier plot was created using a survival curve. Kaplan–Meier curve and logarithmic rank test analyses showed that decreased mRNA levels of *AKR1C1, AKR1C2*, and *AKR6A5* and higher mRNA levels of *AKR7A3* and *AKR1B10* were significantly correlated with better OS, FP, PPS. (*P* < .05) (Fig. 4).

In addition, GC patients with reduced mRNA levels of other AKRs genes, including *AKR1D1, AKR1E2, AKR1B1, AKR6A3*, and *AKR6A9*, were expected to have better OS, FP, and PPS and a better prognosis. It was anticipated that GC patients with higher *AKR1A1, AKR7A2*, and *AKR1C3* mRNA levels would have better OS, FP, and PPS as well as a better prognosis (see Fig. S2, http://links.lww.com/MD/I519, Supplemental Content, which illustrates the Relationship between genes and prognosis).

### 3.4. Mutations of AKRs genes and their relations in GC

**Figure 5.**
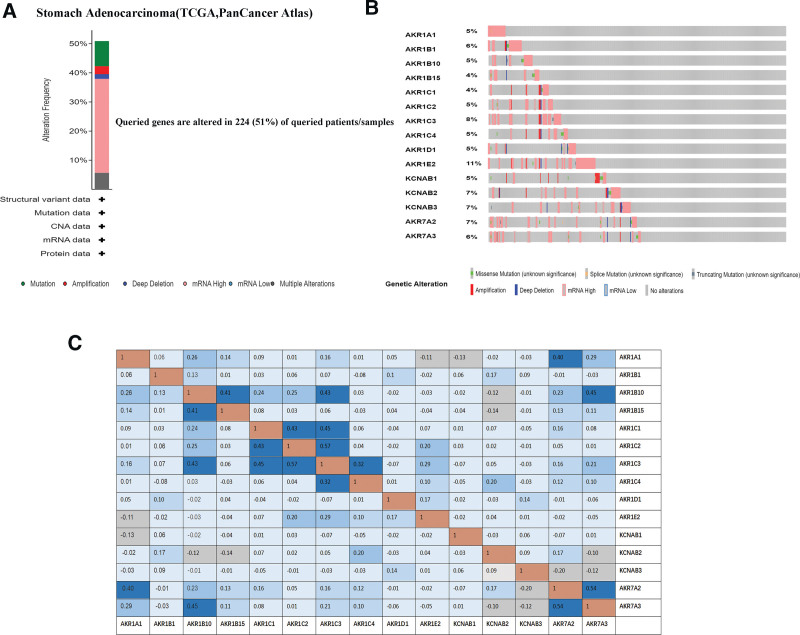
AKRs gene expression and mutation analysis in GC (cBioPortal). AKRs = aldo-keto reductase, GC = gastric cancer.

We analyzed the mutations of AKRs genes and their correlations in GC using the cBioPortal (cBio Cancer Genomics Portal) online tool. *AKR1A1, AKR1B1*, and 13 other genes in STAD (TCGA, PanCancer Atlas) were analyzed using cBioPortal for Cancer Genomics. Among 440 patients with stomach cancer, 224 (51%) were found to be genetically altered (Fig. 5A and B). We also used the cBioPortal online tool for GC (TCGA, PanCancer Atlas) to analyze the mRNA expression levels of the AKRs mutations and computed the correlations between them using Pearson correlation. The results showed a significant positive correlation in the expression of the following AKRs genes: *AKR1A1* with *AKR1B10, AKR7A2*, and *AKR7A3; AKR1B10* with *AKR1A1, AKR1B15, AKR1C1, AKR1C2, AKR1C3, AKR7A2*, and *AKR7A3; AKR1B15* with *AKR1B10; AKR1C1* with *AKR1B10, AKR1C2*, and *AKR1C3; AKR1C2* with *AKR1B10* and *AKR1C3; AKR1C3* with *AKR1B10, AKR1C1, AKR1C2, AKR1C4, AKR1E2*, and *AKR7A3; AKR1C4* with *AKR1C3; AKR1E2* with *AKR1C3; AKR7A2* with *AKR1A1, AKR1B10*, and *AKR7A3*; and *AKR7A3* with *AKR1A1, AKR1B10, AKR1C3*, and *AKR7A2* (Fig. 5C).

### 3.5. Genes that may be regulated by TFs differently in GC

**Figure 6.**
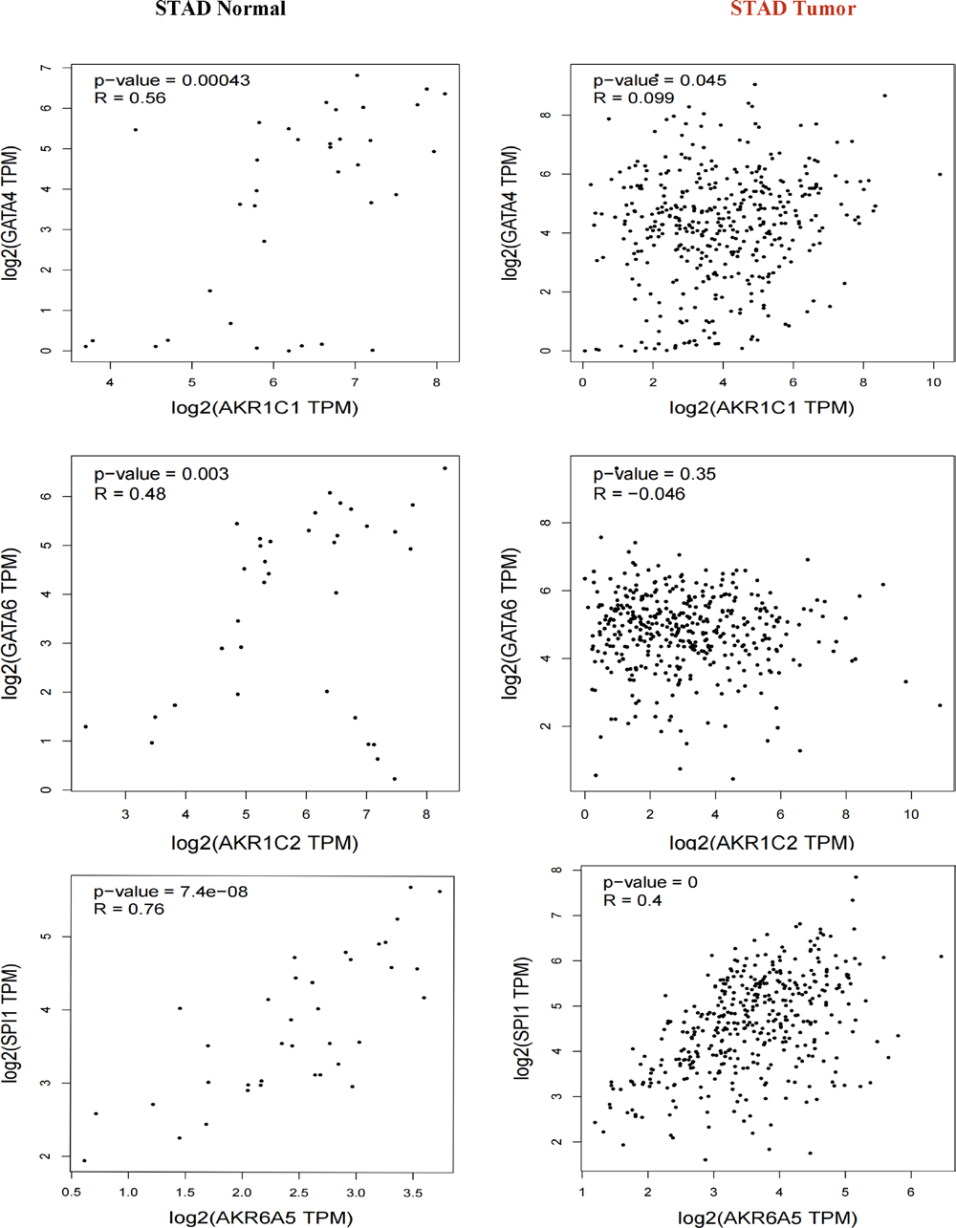
Correlation between the expression of AKRs genes and transcription factors (GEPIA). AKRs = aldo-keto reductase.

Since several differentially expressed AKRs genes are related to prognosis and positively correlated with each other in GC, we explored which TFs can regulate differentially expressed genes in GC. First, the genes that were differentially expressed between GC tissues and healthy tissues were examined. TFs that may regulate *AKR1B10, AKR1C1, AKR1C2, AKR6A5*, and *AKR7A3* in gastric tissues were identified using hTFtarget (a comprehensive database of human TFs and their target regulation) (Table 1). Four genes *SPI1, KLF5, GATA4*, and *GATA6* were identified as genes encoding TFs that may potentially regulate *AKR1B10, AKR1C1, AKR1C2, AKR6A5*, and *AKR7A3* in gastric tissues. The expression of the aforementioned AKRs genes and TF-encoding genes were then correlated using Pearson method. Results show that *GATA4* and *AKR1C1* were statistically significantly correlated in both normal and GC tissues. In normal gastric tissues but not in GC tissue, the expression of *GATA6* was statistically substantially linked with that of *AKR1C2*. In both normal and GC tissues, there was a statistically significant correlation between the expression of *SPI1* and *AKR6A5* (Fig. 6). Importantly, survival analysis revealed that GC patients with low expression of *SPI1* had a better prognosis compared with GC patients with high expression of *SPI1* (see Figs. S1, http://links.lww.com/MD/I518 and S2, http://links.lww.com/MD/I519, Supplemental Content, which illustrates the expression of *SPI1*), which was consistent with the observation that GC patients with low *AKR6A5* expression have a better prognosis than GC patients with high *AKR6A5* expression (Fig. 4).

**Table 1:** TFs that potentially regulate AKRs in gastric tissue (HTFtarget).

Gene	No. of datasets	TF	Tissue	No. of peaks (total/average)	No. of peaks in gene body (total/average)	No. of peaks around TSS (total/average)	The peak close to TSS	The peak with strongest signal
AKR1B10	1	KLF5	Stomach	1/1	1/1	0/0	Chr7,134532214,134532575,17.8,4622,gb,dataset-1899	**Chr7,134532214,134532575,17.8,4622,gb,dataset-1899**
	2	SPI1	Stomach	10/5	5/2	5/2	Chr7,134527318,134527599,5.11,-274,pr,dataset-3179	**Chr7,134532158,134532709,9.09,4566,gb,dataset-3179**
AKR1C1	1	GATA4	Stomach	2/2	2/2	0/0	Chr10,4964850,4965017,4.45,1597,gb,dataset-1496	**Chr10,5020262,5020465,7.86,5700,gb,dataset-1496**
	1	GATA6	Stomach	7/7	7/7	0/0	Chr10,4964791,4965065,8.18,1538,gb,dataset-1520	**Chr10,5020212,5020512,33.3,5695,gb,dataset-1520**
AKR1C2	1	GATA4	Stomach	12/12	11/11	1/1	Chr10,5142186,5142391,6.90,-716,pr,dataset-1497	**Chr10,5071359,5072206,25.8,6302,gb,dataset-1497**
	1	GATA6	Stomach	12/12	10/10	2/2	Chr10,5138024,5138323,12.3,-309,pr,dataset-1520	**Chr10,5020212,5020512,33.3,1147,gb,dataset-1520**
	4	SPI1	Stomach	33/8	29/7	4/1	Chr10,5142260,5142470,3.59,-724,pr,dataset-3126	**Chr10,4981691,4982491,11.5,1527,gb,dataset-3179**
AKR6A5	1	KLF5	Stomach	2/2	2/2	0/0	Chr1,6025342,6025605,9.12,3387,gb,dataset-1899	**Chr1,6026084,6026439,11.6,3461,gb,dataset-1899**
	9	SPI1	Stomach	67/7	58/6	9/1	Chr1,5991470,5992933,36.7,4,pr,dataset-3217	**Chr1,5991457,5992948,45.1,-9,pr,dataset-3148**
AKR7A3	1	KLF5	Stomach	2/2	1/1	1/1	Chr1,19288738,19288949,8.16,301,pr,dataset-1899	**Chr1,19288738,19288949,8.16,301,pr,dataset-1899**
	8	SPI1	Stomach	24/3	14/1	10/1	Chr1,19288632,19289237,7.94,13,pr,dataset-3217	**Chr1,19288770,19289054,11.6196,pr,dataset-3277**

AKRs = aldo-keto reductase, TFs = transcription factors.

### 3.6. The degree of immune infiltration in GC is associated with the expression levels of the AKRs genes

**Figure 7.**
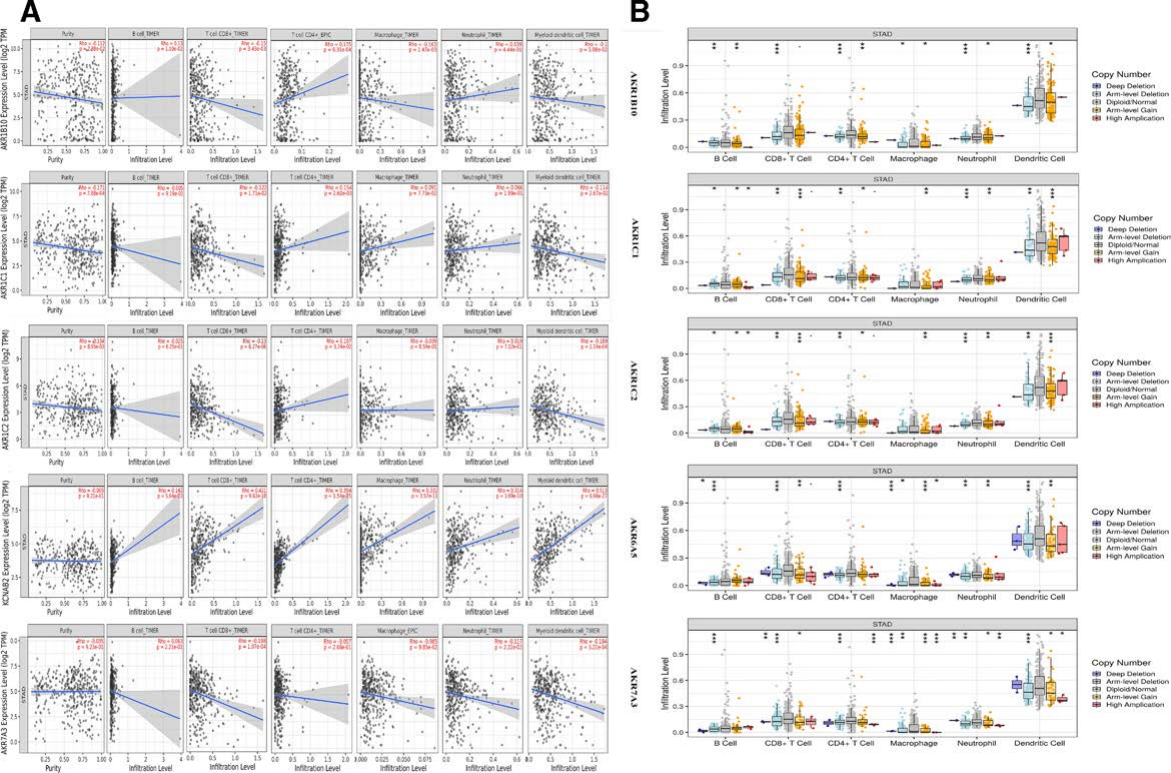
The correlation between differentially expressed AKRs gene status and immune infiltration level in GC (TIMER). (A) The correlation between the expression of differentially expressed AKRs genes and the immune infiltration level in GC. (B) The correlation between differentially expressed AKRs gene copy number variation and the immune infiltration level in GC. *P* value significant codes: 0≤***≤.001≤**.01≤*.05. AKRs = aldo-keto reductase, GC = gastric cancer.

We looked at the relationship between the amount of immune infiltration in STAD and the expression of differentially expressed genes. The results showed that the expression or gene copy number status of *AKR1B10, AKR1C1, AKR1C2, AKR6A5*, and *AKR7A3* in STAD were significantly correlated with the level of immune infiltration of various immune cells, including B cells, CD8 + T cells, CD4 + T cells, macrophages, neutrophils, and dendritic cells (DCs), in the tumor microenvironment (Fig. 7A and B). In addition, the relative immune infiltration level of DCs was the highest among all 6 different types of immune cells in GC, regardless of the condition of AKRs copy number (Fig. 7B).

### 3.7. Prediction of the pathways and roles of the AKRs genes with differential expression in GC

**Figure 8.**
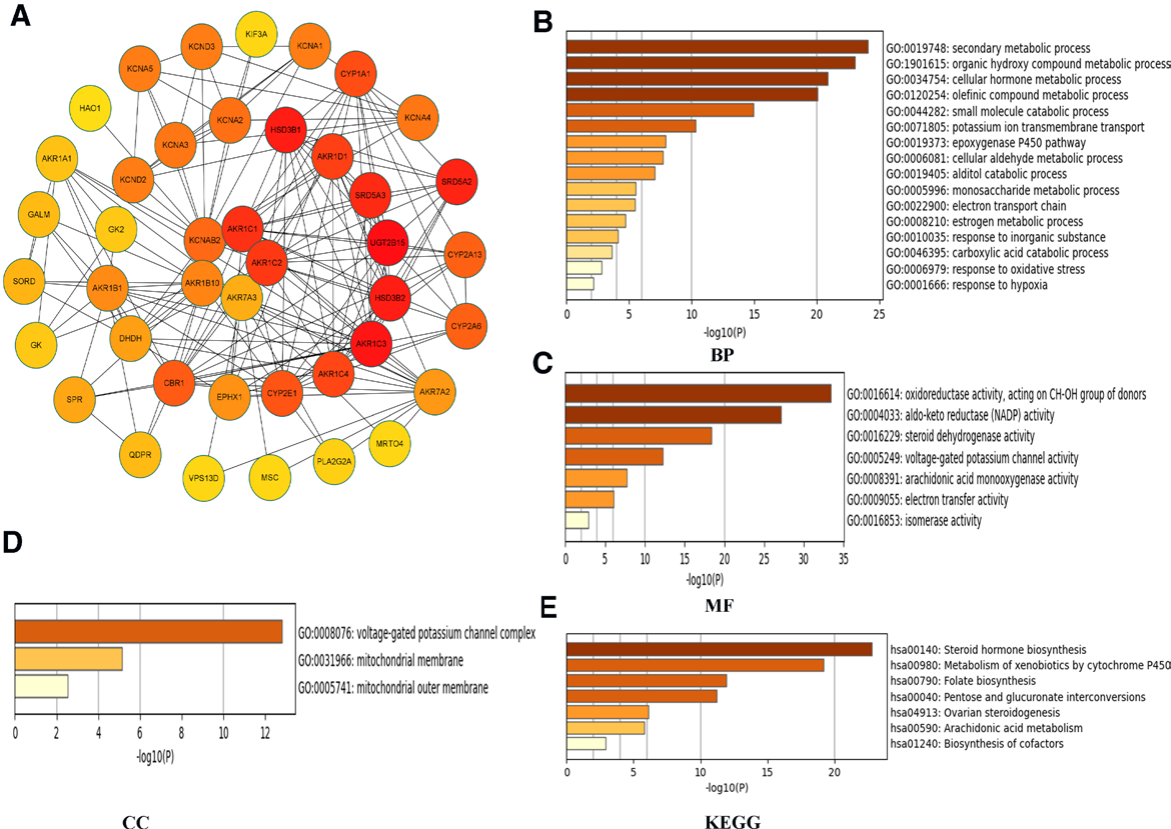
Functional analysis of proteins interacting with differentially expressed genes. Protein–protein interaction networks of AKR genes (A). GO enrichment analysis predicted the functional roles of interacting genes based on 3 categories: biological processes (B), molecular functions (C), and cellular components (D). KEGG signaling pathways (E). AKRs = aldo-keto reductase.

The top 10 candidate proteins that potentially interact with *AKR1B10, AKR1C1, AKR1C2, AKR6A5*, and *AKR7A3* were predicted using the Metascape database. In total, 42 genes, including AKRs genes, were identified in the interaction network (Fig. 8A). GO analysis was performed using these 42 genes. In the biological process category, GO:0019748 (secondary metabolic process), GO:1901615 (organic hydroxy compound metabolic process), GO:0034754 (cellular hormone metabolic process), and GO:0120254 (olefinic compound metabolic process) were significantly enriched (Fig. 8B). In the molecular function category, GO:0016614 (oxidoreductase activity, acting on the CH-OH group of donors) and GO:0004033 (AKRs (NADP) activity) were significantly enriched (Fig. 8C). In the cellular components category, GO:0008076 (voltage-gated potassium channel complex) was significantly enriched (Fig. 8D). Next, on these 42 genes, we ran a KEGG (Kyoto Encyclopedia of Genes and Genomes) pathway enrichment analysis. We found that 7 pathways were significantly enriched (Fig. 8E).

### 3.8. Establishment of a new OS risk scoring model based on human AKRs status for GC patients

**Figure 9.**
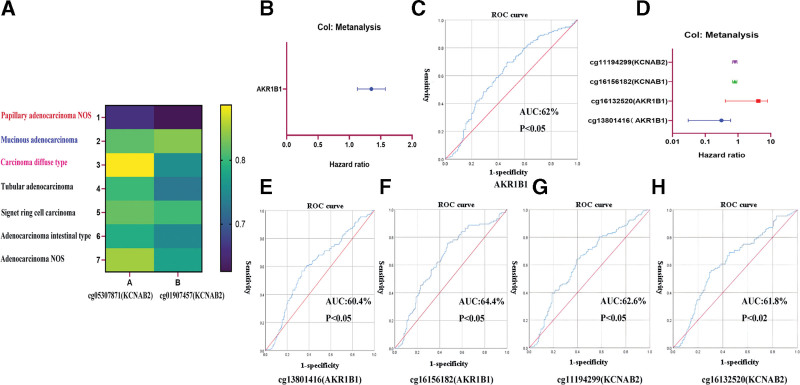
Establishment of a new risk scoring model for GC based on AKRs gene. The 2 sites cg05307871 and cg01907457 of the KCNAB2 gene have the highest differences in methylation levels among different types of GC and the corresponding types (A). The expression levels of AKR1B1 are independent prognostic factors of GC and prognostic accuracy (ROC curve) (B, C). The methylation levels of cg16156182 (KCNAB1), cg11194299 (KCNAB2), cg16132520 (AKR1B1), and 13801416 (AKR1B1) were independent prognostic factors of GC and prognosis (ROC curve) (D–H). AKRs = aldo-keto reductase, GC = gastric cancer.

We analyzed the methylation levels of all 15 AKRs genes in GC using the UCSC database. We found that cg05307871 on the *KCNAB2* gene has the most differential methylation level between the GC subtypes papillary adenocarcinoma NOS and carcinoma diffuse type. The cg01907457 site on the *KCNAB2* gene has the most differential methylation level between the GC subtypes papillary adenocarcinoma NOS and mucinous adenocarcinoma (Fig. 9A). Meta-analysis of survival data, the expression of 15 AKRs genes (Fig. 9B), and methylation levels (Fig. 9D) revealed that high expression of *AKR1B1* has a high hazard ratio (HR) and that high methylation levels of 4 sites in the *KCNAB2, KCNAB1, AKR1B1* genes have a high HR. The ROC curve showed that the area under the curve was above 60% (Fig. 9C, E–H).

## 4. Discussion

Several malignancies have been linked to the AKRs superfamily, and members of the AKRs superfamily have been reported to serve as tumor markers and inhibitors and to play potential roles in therapy, diagnosis, and prognosis.^[16,17]^ However, no further bioinformatics analysis of GC has been performed thus far. In this study, the mRNA expression levels and prognostic values (OS, FP, and PPS) of different AKRs genes in GC were investigated for the first time, relevant information on the immune characteristics of GC was summarized, and TFs related to abnormal expression of AKRs in GC were discussed. Two of the corresponding genes of these TFs are abnormally expressed in GC, so their regulation of AKRs may be tumor-specific and needs to be further explored. However, GO enrichment analysis was performed on the first 50 interacting proteins of the 5 different genes. The protein–protein interaction network further revealed proteins with potential interactions with AKRs, such as *SRD5A3, CYP17A1, UGT2B15*, and *HSD3B1*, which proved that AKRs may act through functional interactions in GC. In addition, we learned that gene methylation levels and AKRs gene expression levels play an important role in GC neutralization and prognosis, and a new prognostic gene model was constructed by COX analysis.

The retinal reductase *AKR1B10* is the most studied member of the AKRs superfamily. *AKR1B10* can change the retinoic acid synthesis pathway and participate in the occurrence and development of tumors.^[18]^ It is extensively expressed in the stomach and intestine epithelial cells, but down-regulated in gastrointestinal carcinoma and inflammatory bowel disease. According to research by Shao et al, GC tissues have considerably less *AKR1B10* expression than healthy stomach tissues. According to the analysis of clinicopathological factors, *AKR1B10* expression had a significant prognostic value in GC patients. Decreased *AKR1B10* expression was found to promote GC cell proliferation and migration. On the contrary, increased expression of *AKR1B10* inhibits the proliferation and migration of GC cells.^[19]^ The ONCOMINE and TCGA datasets used in our investigation demonstrated that the expression of *AKR1B10* in human GC was lower than that in healthy tissues. However, *AKR1B10* was not associated with GC stage. We assessed the predictive significance of *AKR1B10* in GC patients using the Kaplan–Meier Plotter. An improved prognosis was linked to high *AKR1B10* expression. Additionally, we discovered a strong correlation between *AKR1B10* expression and immune cell infiltration levels, including B cells, CD8 + T cells, CD4 + T cells, macrophages, neutrophils, and DCs.

*AKR7A3* is a member of the AKR7A subfamily, also known as aflatoxin reductase, which can prevent aflatoxin B1-induced cytotoxicity and tumor growth.^[20,21]^ Many studies have explored the relationship between *AKR7A3* and cancer. Chow et al found *AKR7A3* inhibits liver cancer.^[10]^ Dai T et al identified 8 differentially expressed AKRs genes in hepatocellular carcinoma. Most AKRs gene dysregulation is negatively correlated with DNA methylation. Regulatory networks with TFs were established. Three key AKRs genes (*AKR1B10, AKR1D1*, and *AKR7A3*) were screened out and a new risk scoring model was established. Finally, upregulation of *AKR1B10* and downregulation of *AKR1D1* and *AKR7A3* were also confirmed in hepatocellular carcinomas compared with adjacent tissues.^[22]^ Little is known about *AKR7A3* expression and its role in GC. In our study, GC tissues have lower *AKR7A3* expression than normal tissues. Additionally, in GC patients, *AKR7A3* expression did not correlate with tumor stage. In all gastric patients, high *AKR7A3* expression was significantly associated with better OS, FP, and PPS.

AKR1Cs include *AKR1C1-4*. These 4 enzymes catalyze NADPH-dependent reduction and are involved in biosynthesis, intermediate metabolism, and detoxification. It has also been suggested that these enzymes are associated with tumors. Altered expression of individual AKR1C genes is associated with the development of prostate, breast, and endometrial cancers. Chang et al clarified the specific role of *AKR1C1* in metabolic reprogramming and suggested that *AKR1C1* may serve as a new therapeutic target for NSCLC treatment. A number of illnesses, including prostate cancer, breast cancer, endometrial cancer, ovarian cancer, cervical cancer, lung cancer, NSCLC, and preterm labor, have been linked to abnormal *AKR1C1* expression (delivery time problem).^[23–26]^ Tian Y et al found that *AKR1C3* expression in castration-resistant prostate cancer was higher than that in prostate cancer, suggesting that *AKR1C3* expression can be used as a promising biomarker for evaluating the progression of prostate cancer.^[27]^ Phoo NLL et al used transcriptome analysis to reveal that *AKR1C1* and *1C3* regulate Signet ring cell gastric carcinoma cisplatin resistance through redox-dependent autophagy.^[28]^ We used the database to analyze the expression and prognostic value of the AKR1C family in GC.

The present work offers insight into the variability and complexity of the molecular biological aspects of GC by methodically analyzing the expression and prognostic value of AKRs in GC. Our findings imply that elevated *AKR6A5 (KCNAB2*) expression in GC may be crucial to GC. Studies have indicated that the main function of parietal cells in gastric mucosa is to participate in gastric acid secretion, and these activities may also be closely related to the occurrence of GC.^[29]^ H^+^ secreted by gastric wall cells is produced by intracellular hydrolysis. The energy released by H^+^-K^+^ ATPase on the secretory tubule for every molecule of ATP hydrolyzed drives 1 H^+^ from the cytoplasm into the secretory tubule and 1 K^+^ from the secretory tubule into parietal cells. Under the action of the proton pump, H^+^ is actively transported from the cytoplasm to secretory tubules. K^+^ enters human cells and then enters the secretory tubule lumen through the K^+^ channel, while Cl^−^ enters the secretory tubule lumen through the Cl^−^ channel, forms HCl with H^+^, and then enters the gastric lumen.^[30]^ The combination of rapid KCl influx and subsequent H^+^-K^+^ ATPase-driven hydrogen and potassium ion exchange allows the efficient accumulation of acid in secretory tubules and vesicles, while the intracytoplasmic hydrogen ion levels outside the secretory tubules were lower and had higher pH.^[31]^ By searching public databases, we discovered that the voltage-gated potassium channel subunit-2 (*KCNAB2*) is expressed differently in GC tissues compared to normal tissues and that GC patients have a worse prognosis when their expression is high. Therefore, we considered whether *KCNAB2* affects the K^+^ channel and hence the concentrations of K^+^ and H^+^, as well as GC prognosis, which remains to be further explored. In addition, high *AKR6A5* expression can also be used as a molecular marker to spot GC patients and possible targets for treatment. Among the established prognostic gene models, *KCNAB2* methylation showed the largest difference among different types of GC and was strongly correlated with prognosis. In addition, *AKR7A3* and *AKR1B1* are potential prognostic markers for improving GC survival, and 5 differentially expressed genes have a potential value in predicting immune infiltration. We also found that among several TFs regulating differentially expressed genes, the gene encoding *SPI1* was upregulated in GC, and *AKR6A5* was also upregulated in GC. Moreover, *SPI1* expression was correlated with *AKR6A5* expression. Therefore, the relationship between *SPI1* and *AKR6A5* is also worth further study. In conclusion, we hope that our work will contribute to existing knowledge, improve clinical treatment regimens, provide more guiding theories to utilize the AKRs superfamily and potential targets for GC diagnosis and treatment, and improve outcomes for GC patients.

## Author contributions

**Conceptualization:** Yujin Zhou.

Data curation: Quan Liu.

Formal analysis: Wenjing Li, Hui Gong.

Funding acquisition: Yifan Li.

Project administration: Dixian Luo.

Supervision: Yi Lin.

Visualization: Yi Lin.

Writing – original draft: Yujin Zhou, Wenjing Li, Quan Liu, Hui Gong, Yifan Li, Dixian Luo.

Writing – review & editing: Yujin Zhou, Yi Lin, Yi Lin, Wenjing Li, Quan Liu, Hui Gong, Yifan Li, Dixian Luo.

## Supplementary Material




