# Supplementary material for: Expression of AKRs superfamily and prognostic in human gastric cancer
Source: Medicine (Baltimore). 2023 Feb 22;102(8):e33041. doi: 10.1097/MD.0000000000033041 (PMC11309706; doi:10.1097/MD.0000000000033041)
Supplement: Supplementary file 1 [file medi-102-e33041-s001.pdf]

Supplementary Figure 2

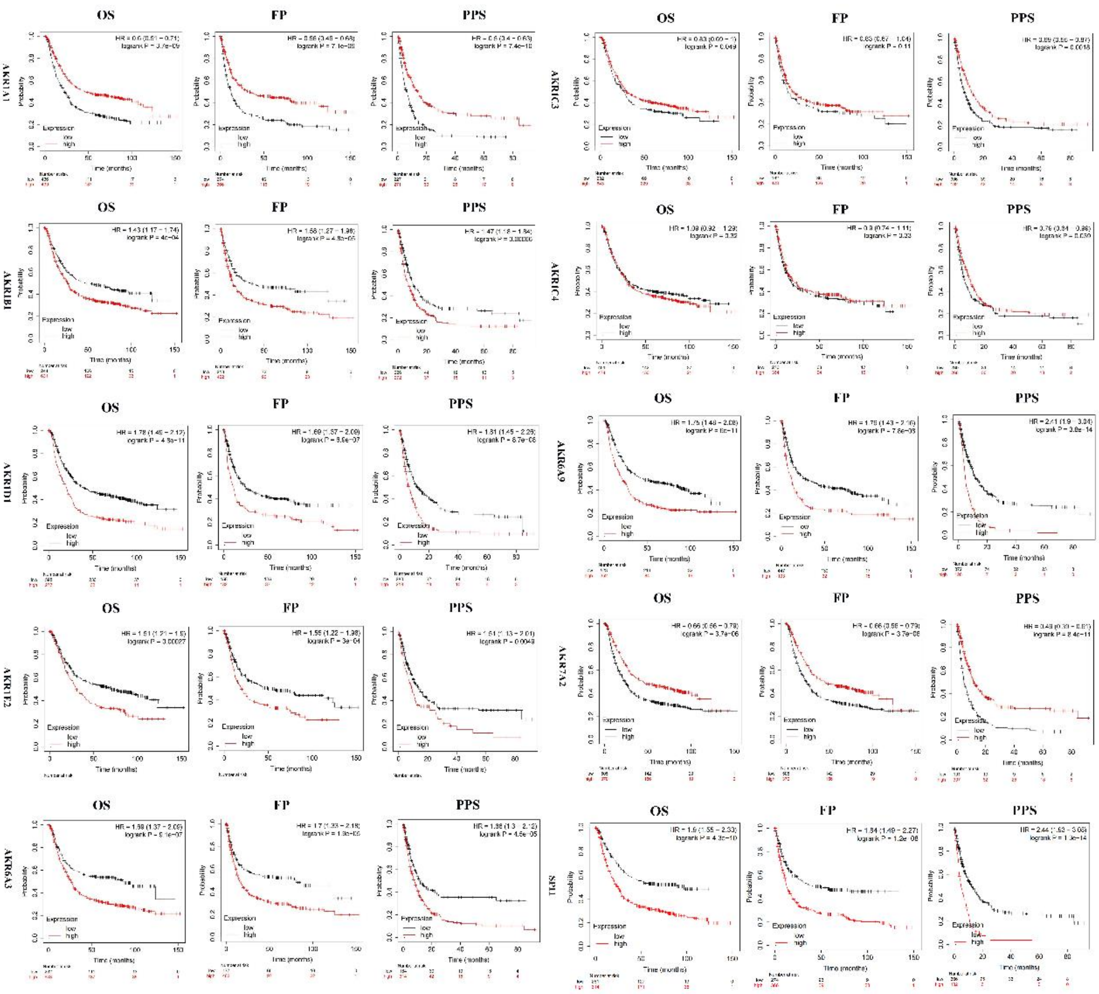

Figure S2: Prognostic values of mRNA levels in GC patients (Kaplan-Meier Plotter).
