# Supplementary material for: Expression of AKRs superfamily and prognostic in human gastric cancer
Source: Medicine (Baltimore). 2023 Feb 22;102(8):e33041. doi: 10.1097/MD.0000000000033041 (PMC11309706; doi:10.1097/MD.0000000000033041)
Supplement: Supplementary file 2 [file medi-102-e33041-s002.pdf]

Supplementary Figure 1

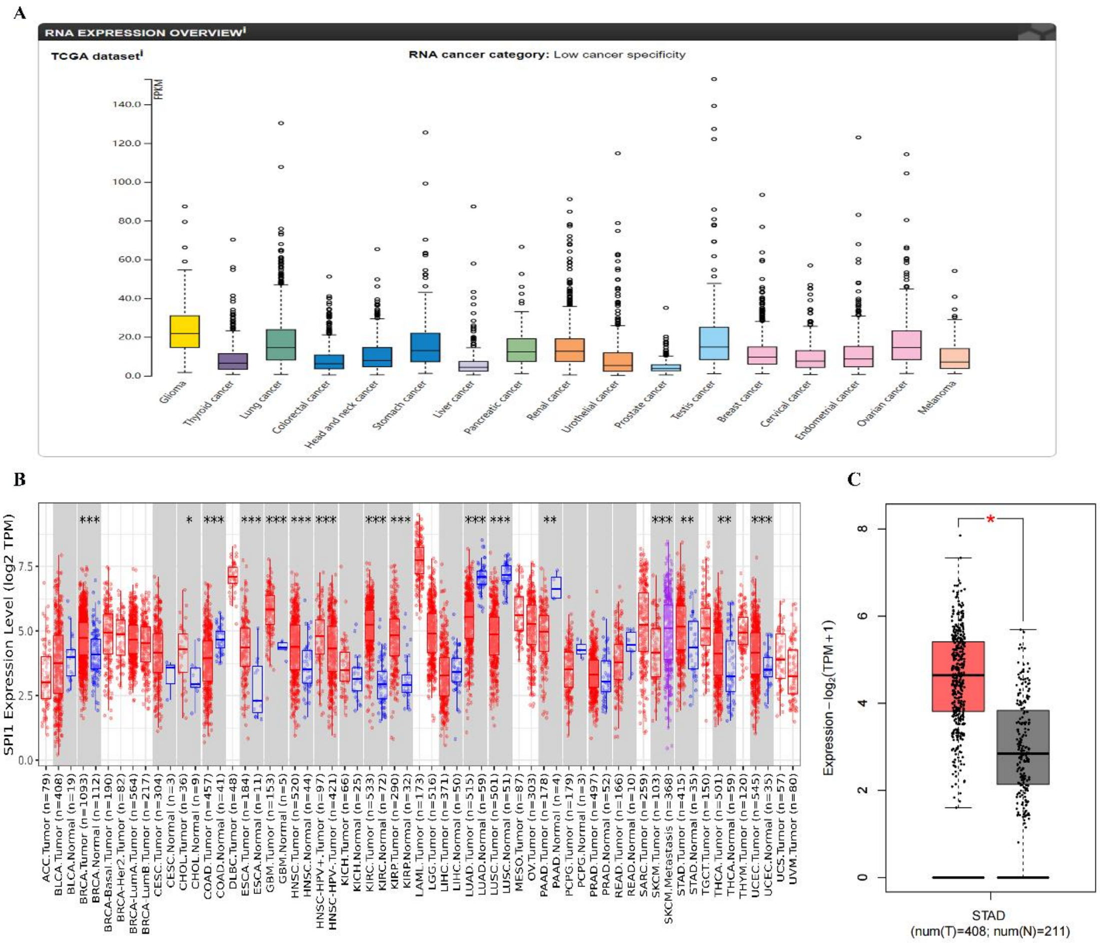

Figure S1: The gene expression levels of *SPI1* in various types of human cancer. HPA results (A) . TIMER results (B). GEPIA results (C). *P*-value significant codes:0⩽***⩽0.001⩽**⩽0.01⩽*⩽0.05
